# Mitapivat-Associated Rib Fracture in a Hemolytic Anemia Patient

**DOI:** 10.7759/cureus.55658

**Published:** 2024-03-06

**Authors:** Yasser Abouelkheer, Luisa Ladel, Daniel Boxer, Seaf Shafique

**Affiliations:** 1 Internal Medicine, Norwalk Hospital/Yale University, Norwalk, USA; 2 Hematology and Oncology, Norwalk Hospital, Norwalk, USA; 3 Internal Medicine, Norwalk Hospital, Norwalk, USA

**Keywords:** adverse event, case report, fracture, anemia, hemolytic, hereditary, mitapivat

## Abstract

Hereditary hemolytic anemia associated with pyruvate kinase deficiency is a rare hematological disorder that affects the glycolic pathway within red blood cells. The standard of care includes splenectomy, transfusions, and hematopoietic stem cell transplantation. However, these treatments can lead to common iatrogenic side effects such as infections, surgical complications, and iron overload. The novel drug therapy Mitapivat has shown promising results in terms of both efficacy and safety, but it can cause rare side effects such as fractures. In this report, we present the case of a 75-year-old female with hereditary hemolytic anemia caused by pyruvate kinase deficiency who suffered rib and vertebral body fractures after the initiation of Mitapivat. Screening for key risk factors of bone mineral disease can help identify patients who are at higher risk of developing fractures before starting therapy. In the future, gene therapy may provide an alternative treatment option for patients with hereditary hemolytic anemia with metabolic bone disorders.

## Introduction

Hereditary hemolytic anemia is characterized by early red blood cell (RBC) destruction, most commonly due to inherited metabolic abnormalities such as pyruvate kinase deficiency (PKD). In PKD, the deficiency of PK in RBCs affects the conversion of phosphoenolpyruvate to pyruvate, ultimately impairing adenosine triphosphate (ATP) production and the maintenance of the cell membrane [1]. As a result, RBCs are destroyed in the spleen, leading to extravascular hemolysis [1,2]. PKD is an autosomal recessive disorder that results in different levels of severity depending on the enzyme isoform, expression levels, and inheritance pattern (homozygous vs. heterozygous). For example, pediatric patients with homozygous PKD can experience severe and frequent episodes of hemolysis, leading to transfusion dependence [2]. Conversely, adults with heterozygous disease may experience isolated episodes of anemia following physiological stress or liver failure associated with iron overload [2].

The confirmation of PKD diagnosis involves genetic studies, which are typically conducted as part of neonatal hyperbilirubinemia workup in perinatal cases or unexplained severe anemia in adults [1,2]. Splenectomy and hematopoietic stem cell transplantation can be beneficial for patients with transfusion-resistant disease, but these interventions are associated with complications such as infections, thrombocytosis, graft versus host disease, and surgical risks. Therefore, new research studies and clinical trials have focused on developing novel drug therapies that restore enzyme function in PKD [3].

Mitapivat is a small-molecule drug that has been designed as an allosteric activator of the PK enzymes (both wild-type and mutant), resulting in significant increases in PK activity and levels of ATP in vitro [3]. Preclinical studies show that Mitapivat reduces anemia, markers of hemolysis, iron overload, and spleen weight [3]. Clinical trials, such as DRIVE-PK and ACTIVATE, have demonstrated reduced transfusion burden and an excellent safety profile in adults with PKD [3-5]. However, a key feared side-effect of Mitapivat is the development of fractures due to off-target aromatase inhibition in a PKD patient population that is predisposed to osteopenia and osteoporosis [3]. In the phase 3, randomized, double-blind, placebo-controlled trial ACTIVATE, out of the 40 patients who received Mitapivat, 1 patient developed a rib fracture [4].

## Case presentation

**Figure 1:**
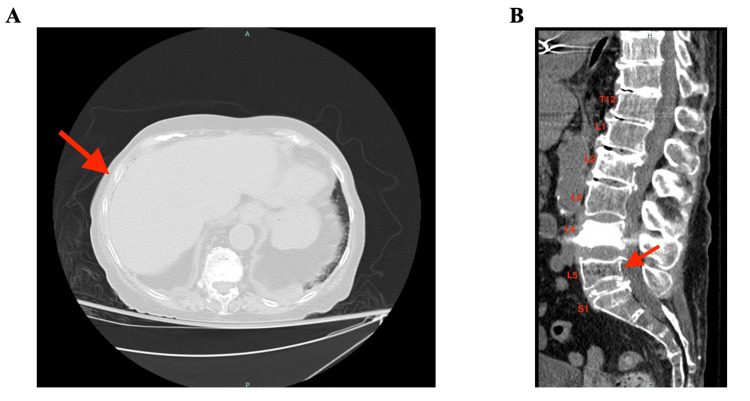
Fractures are a rare side effect of Mitapivat. A 75-year-old female with hereditary hemolytic anemia due to pyruvate kinase deficiency developed a rib and vertebral body fracture 18 days after starting Mitapivat. A: CT chest demonstrating nondisplaced fracture at the lateral right ninth rib. B: CT spine demonstrating multiple compression fractures, including L2, L4, and L5 vertebrae. L2 and L4 findings are consistent with chronic fractures with known kyphoplasty at L4. Radiological findings of L5 are consistent with a new compression fracture.

A 75-year-old female, with a history of hereditary hemolytic anemia due to pyruvate kinase deficiency, liver-biopsy-proven secondary iron overload, and L4 compression fracture post-kyphoplasty on long-term steroids, presented to the emergency department 18 days after starting Mitapivat, with new lower-back and right-sided chest pain. The patient reported experiencing moderate pain for 48 hours that worsened with movement and was partially relieved by simple analgesia. No traumatic events occurred prior to the onset of pain. The pain was not associated with new motor or sensory deficits, changes in bowel or urinary habits, fevers, or weight loss. On presentation, she was afebrile and hemodynamically stable. Physical examination was remarkable for point tenderness at the right chest laterally and the lower back at the lumbar region. Laboratory investigations were significant for a stable hemoglobin level of 8.6 g/dL, with a mean corpuscular volume of 114 fL and red blood cell distribution width of 25.9%. Computed tomography (CT) imaging demonstrated a nondisplaced right ninth rib fracture laterally and multiple compression fractures, including L2, L4, and L5 vertebral bodies (Figure 1). The patient was admitted to the hospital for pain management, percutaneous vertebral augmentation, and physical therapy. After shared-decision making, Mitapivat was discontinued, and the case was reported for medication side effects.

## Discussion

Hereditary hemolytic anemia due to PKD is a complex genetic disease that leads to systemic complications. The current standard of care involves splenectomy, transfusions, and hematopoietic stem cell transplantation, but it is often associated with multiple iatrogenic side effects, including iron overload, infections, and surgical complications. Mitapivat is a novel drug that shows promising clinical safety and efficacy profiles, opening new avenues for drug innovation in this disease [4,5]. However, rare side effects of this treatment can lead to severe comorbidities and functional decline in patients. In this case, the patient experienced bone fractures following the initiation of Mitapivat, which required the discontinuation of the drug and resulted in a hospital admission. This medication side-effect is therefore classified as a Level 4 on the Hartwig’s Severity Assessment Scale [6]. Additionally, it has caused functional decline, psychosocial stress, and financial burden, leading to short-term and long-term negative health outcomes.

Patients with hereditary hemolytic anemia are predisposed to osteopenia and osteoporosis and, consequently, are at higher risk for fractures [3]. In addition, patient populations with comorbidities that exacerbate the risk of these bone mineral diseases represent a high-risk group for fractures. Mitapivat-associated fractures can, therefore, occur at higher rates within subpopulations with multiple risk factors, resulting in significant functional decline. Identifying high-risk patients by screening for risk factors associated with bone disease and discussing the risks and benefits of treatment with patients can help reduce the risks associated with Mitapivat treatment. Close monitoring is also necessary after the initiation of this drug to minimize comorbidities and enhance the functional status of patients with hereditary hemolytic anemia.

Gene therapy offers a potential treatment for PKD, providing an alternative to the current standard of care. Preclinical studies using lentiviral vectors in mouse models have demonstrated significant phenotypic correction with a reliable safety profile [7]. Additionally, a global phase 1 study using hematopoietic stem and progenitor cells transduced with lentiviral vectors has demonstrated normalization of hemoglobin levels and an enhanced quality of life [8]. These studies offer new perspectives on the management of hemolytic anemia with PKD and advance our understanding of the unexplored territories of gene therapy.

## Conclusions

Mitapivat is a novel drug therapy for patients with hereditary hemolytic anemia. It has demonstrated excellent efficacy and safety profile in clinical trials. However, the rare side effect of bone fractures can result in serious comorbidities with severe functional decline. Screening for risk factors related to bone mineral disease can help identify high-risk patients who should avoid Mitapivat treatment. Alternative treatments in development such as gene therapy may provide a safer profile for patients with hereditary hemolytic anemia and metabolic bone disorders.

## References

[1] Al-Samkari H, Van Beers EJ, Kuo KH (2020). The variable manifestations of disease in pyruvate kinase deficiency and their management. Haematologica.

[2] Fattizzo B, Cavallaro F, Marcello AP, Vercellati C, Barcellini W (2022). Pyruvate kinase deficiency: current challenges and future prospects. J Blood Med.

[3] Al-Samkari H, van Beers EJ (2021). Mitapivat, a novel pyruvate kinase activator, for the treatment of hereditary hemolytic anemias. Ther Adv Hematol.

[4] Al-Samkari H, Galactéros F, Glenthøj A (2022). Mitapivat versus placebo for pyruvate kinase deficiency. N Engl J Med.

[5] Grace RF, Rose C, Layton DM (2019). Safety and efficacy of mitapivat in pyruvate kinase deficiency. N Engl J Med.

[6] Petrova G, Stoimenova A, Dimitrova M, Kamusheva M, Petrova D, Georgiev O (2017). Assessment of the expectancy, seriousness and severity of adverse drug reactions reported for chronic obstructive pulmonary disease therapy. SAGE Open Med.

[7] Garcia-Gomez M, Calabria A, Garcia-Bravo M (2016). Safe and efficient gene therapy for pyruvate kinase deficiency. Mol Ther.

[8] Shah AJ, López Lorenzo JL, Sevilla J (2022). Lentiviral-mediated gene therapy for adults and children with severe pyruvate kinase deficiency: results from an ongoing global phase 1 study. Blood.

